# TET1 Interacts Directly with NANOG via Independent Domains Containing Hydrophobic and Aromatic Residues

**DOI:** 10.1016/j.jmb.2020.10.008

**Published:** 2020-11-20

**Authors:** Raphaël Pantier, Nicholas Mullin, Elisa Hall-Ponsele, Ian Chambers

**Affiliations:** Centre for Regenerative Medicine, Institute for Stem Cell Research, School of Biological Sciences, University of Edinburgh, 5 Little France Drive, Edinburgh EH16 4UU, Scotland, United Kingdom

**Keywords:** protein–protein interactions, embryonic stem cells, chromatin, pluripotency, enhancers, DSBH, double stranded beta helix domain, ESC, embryonic stem cell, NID, NANOG-interacting domain, OGT, O-linked N-acetylglucosamine transferase, TET, ten-eleven-translocation, WR, tryptophan-repeat

## Abstract

The DNA demethylase TET1 is highly expressed in embryonic stem cells and is important both for lineage commitment, and reprogramming to naïve pluripotency. TET1 interacts with the pluripotency transcription factor NANOG which may contribute to its biological activity in pluripotent cells. However, how TET1 interacts with other proteins is largely unknown. Here, we characterise the physical interaction between TET1 and NANOG using embryonic stem cells and bacterial expression systems. TET1 and NANOG interact through multiple binding sites that act independently. Critically, mutating conserved hydrophobic and aromatic residues within TET1 and NANOG abolishes the interaction. On chromatin, NANOG is predominantly localised at ESC enhancers. While TET1 binds to CpG dinucleotides in promoters using its CXXC domain, TET1 also binds to enhancers, though the mechanism involved is unknown. Comparative ChIP-seq analysis identifies genomic loci bound by both TET1 and NANOG, that correspond predominantly to pluripotency enhancers. Importantly, around half of NANOG transcriptional target genes are associated with TET1-NANOG co-bound sites. These results indicate a mechanism by which TET1 protein may be targeted to specific sites of action at enhancers by direct interaction with a transcription factor.

## Highlights

• TET1 and NANOG interact via multiple independent binding regions.
• TET1 and NANOG interactions are mediated by aromatic and hydrophobic residues.
• TET1 residues that bind NANOG are highly conserved in mammals.
• Co-localisation of TET1 and NANOG on chromatin is enriched at NANOG target genes.
• NANOG and TET1 have regulatory roles in maintaining and reprogramming pluripotency.

## Graphical abstract

**Figure:**
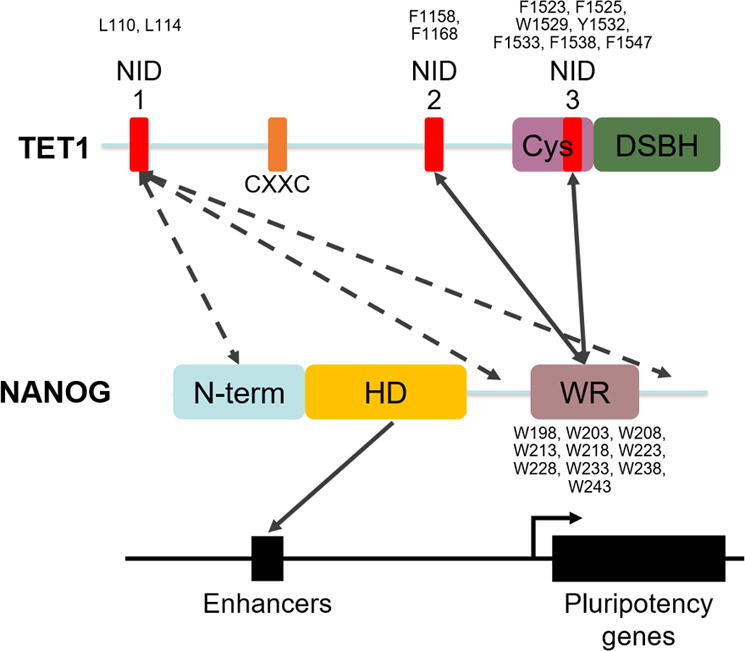

## Introduction

Ten-eleven-translocation (TET) family proteins are responsible for active DNA demethylation by oxidation of 5-methylcytosine1., 2. and play important roles during embryonic development and various physiological processes.^3^ TET proteins contribute to DNA demethylation in naïve embryonic stem cells (ESCs),4., 5., 6., 7. in particular at enhancers.8., 9., 10., 11., 12., 13. TET protein activity is required both for proper differentiation14., 15. and reprogramming to pluripotency.16., 17., 18. TET1 is the most highly expressed TET family protein both in pluripotent cells and during early development.19., 20., 21. TET1 predominantly binds to promoters via its N-terminal CXXC domain which recognises unmethylated CpG dinucleotides.22., 23., 24., 25. TET1 binding at enhancers in ESCs26., 27., 28. could be mediated by interactions with the pluripotency factors NANOG, PRDM14, OCT4 and SOX2.29., 30., 31., 32. Interestingly, co-expression of TET1 and NANOG in pre-iPS cells synergistically enhances reprogramming to pluripotency.^29^ However, how TET1 might be recruited to chromatin via protein–protein interactions remains poorly understood with little known about the residues involved in protein binding.

Here, the interaction between TET1 and the pluripotency factor NANOG was characterised in ESCs. Co-immunoprecipitations using an array of TET1 truncations and mutants uncovered novel regions involved in protein–protein interactions, both within and outwith the well characterised catalytic domain. Furthermore, alanine mutagenesis identified single residues that show high evolutionary conservation and that contribute to the interaction of TET1 with NANOG. Comparison of TET1 and NANOG ChIP-seq datasets identified genomic loci that are putatively regulated by the TET1-NANOG complex.

## Results

### The TET1 N-terminus interacts directly with NANOG via the evolutionary conserved residues L110 and L114

**Figure 1:**
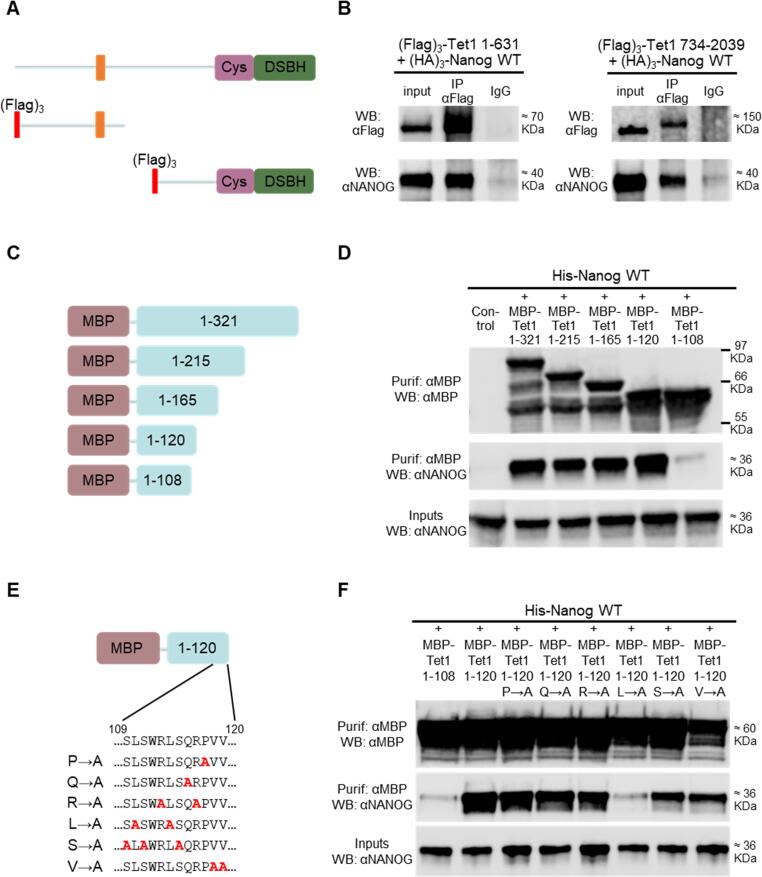
TET1 contains independent NANOG-binding regions. (a) and (b) Co-immunoprecipitations of non-overlapping (Flag)_3_-TET1 N- and C-terminal constructs (a) with (HA)_3_-NANOG from E14/T ESCs. (b) Immunoblots were probed with the antibodies indicated on the left (representative images, *n* = 2). (c) and (d) Co-purification of MBP-tagged TET1 N-terminal constructs with (His)_6_-NANOG from *E. coli*. (c) Fragments of the TET1 N-terminus are shown in the context of full length TET1. (d) Immunoblots were probed with the antibodies indicated on the left (representative images, *n* = 3). (e) and (f) Co-purification of (His)_6_-NANOG with alanine substitution mutants of MBP-TET1 (1–120) from *E. coli*. (e) Alanine substitution mutants of TET1 (109–120) are shown in the context of full length TET1. (f) Immunoblots were probed with the antibodies indicated on the left (representative images, *n* = 3).

The TET1 protein expressed in mouse ESCs is composed of 2039 residues. TET1 is characterised by an evolutionary conserved C-terminal catalytic domain, that can be subdivided into a cysteine rich region (residues 1367–1550) and a double stranded beta helix domain (DSBH) (residues 1551–2039) (Figure S1(a)). TET1 also possesses a CXXC domain (residues 567–608), a DNA binding region.^33^ NANOG is a 305 amino acids transcription factor comprising a N-terminal domain (residues 1–95), a DNA binding homeodomain (residues 96–155) and a C-terminal region containing a tryptophan-repeat (WR) (residues 199–243) (Figure S1(a)). TET1 has been identified as a NANOG-binding protein by independent affinity purification-mass spectrometry analyses.29., 30. We therefore analysed the interaction between endogenous TET1 and NANOG in pluripotent cells using nuclear protein extracts from *Tet1-(Flag)_3_* ESCs^21^ immunoprecipitated with an anti-Flag antibody. Relative to controls from E14Tg2a ESCs which showed only background binding, FLAG immunoprecipitates from *Tet1-(Flag)_3_* ESCs were strongly enriched for NANOG (Figure S1(b)). This confirms that NANOG and TET1 proteins interact in ESCs when expressed at endogenous levels. Notably, TET1 protein in immunoprecipitates migrates slower than input material on immunoblots; a phenomenon previously reported.34., 35., 36. Next, to determine whether TET1 interacts with NANOG via the TET1 N- or C-terminus, two large (Flag)_3_-tagged TET1 fragments 1–631 and 734–2039 were cloned and expressed in ESCs (Figure 1(a)), together with NANOG. Following TET1 immunoprecipitation, NANOG was co-immunoprecipitated with both constructs (Figure 1(b)). As TET1 1–631 and 734–2039 do not contain overlapping residues, these results suggest that TET1 contains at least two NANOG-interacting domains (NIDs) that function independently.

To begin to explore how NANOG binds to the TET1 N-terminus, (Flag)_3_-tagged TET1 fragments 1–321, 1–215 and 1–108 were cloned and expressed in ESCs (Figure S1(c)), together with NANOG. NANOG was co-immunoprecipitated with TET1(1–321) and TET1(1–215) but not TET1(1–108) (Figure S1(d)). To home in on the NANOG-interacting domain within the TET1 N-terminus and to determine whether the interaction between the TET1 N-terminus and NANOG was direct, both (His)_6_-tagged NANOG and several MBP-tagged TET1 fragments (1–321, 1–215, 1–165, 1–120, 1–108) were cloned into IPTG-inducible plasmids and expressed in *Escherichia coli* (Figure 1(c)). MBP-TET1 fragments purified using an amylose resin were examined for co-purifying NANOG by immunoblotting. NANOG co-purified with all TET1 fragments, except TET1(1–108) which showed a dramatically decreased interaction with NANOG (Figure 1(d)). Importantly, these experiments confirmed a direct physical interaction and narrowed down the first NANOG-interacting domain (NID 1) to 11 residues (109–120). Protein alignments showed that NID 1 is highly conserved among mammals, indicating a selective pressure for the conservation of these TET1 residues (Figure S1(e)). However, residues 109–120 of TET1 do not align with TET2 or TET3 proteins (data not shown). To identify which residues are responsible for binding to NANOG, the MBP-TET1 1–120 plasmid construct was modified by alanine substitution of specific amino acids (proline, glutamine, arginine, leucine, serine and valine) within residues 109–120 (Figure 1(e)). The binding of NANOG to each mutant construct was assessed following bacterial expression and TET1 purification. Strikingly, only the L→A mutant (L110A, L114A) showed a decreased interaction with NANOG, which was reduced to a similar extent as the negative control TET1 1–108 (Figure 1(f)). Together, these data indicate that one or both of the two evolutionary conserved leucine residues (L110 and/or L114) are in direct physical interaction with NANOG.

**Figure 2:**
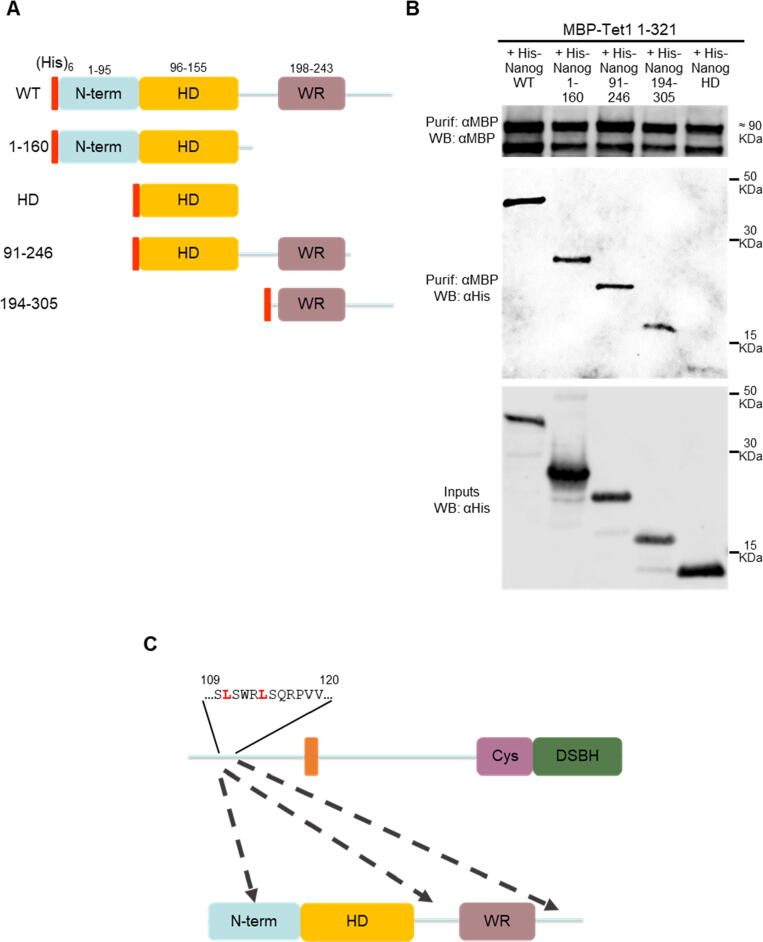
Multiple NANOG regions interact with the TET1 N-terminal domain. (a) and (b) Co-purification of MBP-TET1 1–321 with (His)_6_-NANOG constructs (a) from *E. coli*. (b) Immunoblots were probed with the antibodies indicated on the left (representative images, *n* = 2). (c) Diagram of the interactions between the TET1 NID 1 and NANOG, highlighting critical leucines (red). Dashed arrows indicate potential TET1-interacting regions in NANOG.

To determine which regions of NANOG interact directly with the TET1 N-terminus, MBP-TET1 1–321 was co-expressed with several (His)_6_-tagged NANOG truncations in *E. coli* (Figure 2(a)). Surprisingly, the TET1 N-terminus interacted with three out of four NANOG truncations: 1–160, 91–246, and 194–305 (Figure 2(b)). However, the TET1 N-terminus showed no interaction with the NANOG homeodomain (Figure 2(b)). Moreover, while the NANOG WR interacts with MBP-SOX2 (positive control^30^), MBP-TET1 1–321 showed no physical interaction with the NANOG WR (Figure S2). Collectively, these results indicate that the TET1 N-terminus interacts with several independent sites on NANOG, and independently of its two most characterised domains: the homeodomain and the WR (Figure 2(c)).

### The TET1 C-terminus contains two domains that bind NANOG via aromatic interactions

**Figure 3:**
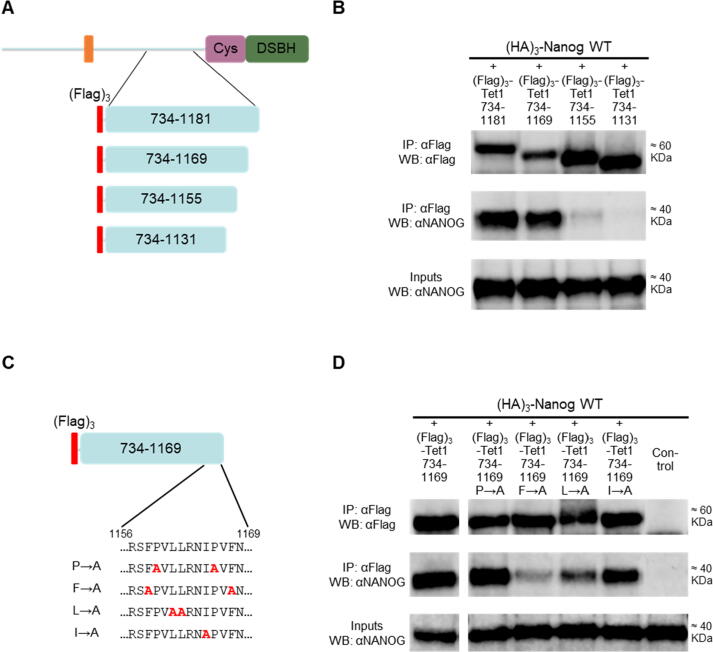
The NANOG interaction domain 2 of TET1 binds NANOG using phenylalanine residues. (a) and (b) Co-immunoprecipitation of the (Flag)_3_-TET1 constructs (a) with (HA)_3_-NANOG from E14/T ESCs. (b) Immunoblots were probed with the antibodies indicated on the left (representative images, *n* = 3). (c) and (d) Co-immunoprecipitation of alanine substitution mutants of (Flag)_3_-TET1(734–1169) with (HA)_3_-NANOG from E14/T ESCs. (c) Alanine substitutions of the amino acids within residues 1156–1169. (d) Immunoblots were probed with the antibodies indicated on the left (representative images, *n* = 2).

Following the initial observation that TET1 contains >1 independent NANOG-interacting domains (Figure 1(b)), the TET1 C-terminus was analysed to identify the NANOG-interacting residues. Regions of TET1 extending from 734 to varying degrees towards the Cys domain were expressed together with NANOG in ESCs (Figures 3(a) and S3(a)). TET1 fragments containing truncations up to residue 1181 (734–1229, 734–1202, 734–1181) were able to bind NANOG, while further C-terminal truncations (734–1155 and 734–1131) abolished interaction with NANOG (Figure S3(a) and (b)). Analysis of a further truncation (734–1169) narrowed down the second NANOG-interacting domain of TET1 to residues 1156–1169 (NID 2) (Figure 3(a) and (b)). A construct containing NID 2 did not interact with NANOG when co-expressed in *E. coli* (Figure S3(c) and (d)) suggesting that either the interaction observed in ESCs is indirect, or that a direct interaction dependent on post-translational modifications and/or protein folding could not be reproduced in bacteria. However, this interaction does not depend on phosphorylation. While treatment of ESC protein extracts with phosphatase affected the mobilities of both TET1 and NANOG proteins, TET1(734–1169) retained the capacity to bind NANOG (Figure S3(e)).

Residues 1156–1169 of mouse TET1 (NID 2) have a high similarity to sequences of TET1 proteins from other mammals, with phenylalanine 1158 strictly conserved (Figure S3(f)). This region did not align with TET2 or TET3 proteins (data not shown). To identify residues that bind NANOG, the expression plasmid encoding TET1 734–1169 was modified by alanine substitution of specific amino acids (proline, phenylalanine, leucine and isoleucine) within residues 1156–1169 (Figure 3(c)). The F→A mutant (F1158A, F1168A) was the only mutant that showed a decreased interaction with NANOG (Figure 3(d)). Together, these results indicate that phenylalanine 1158 and/or 1168 are critical for NANOG binding.

**Figure 4:**
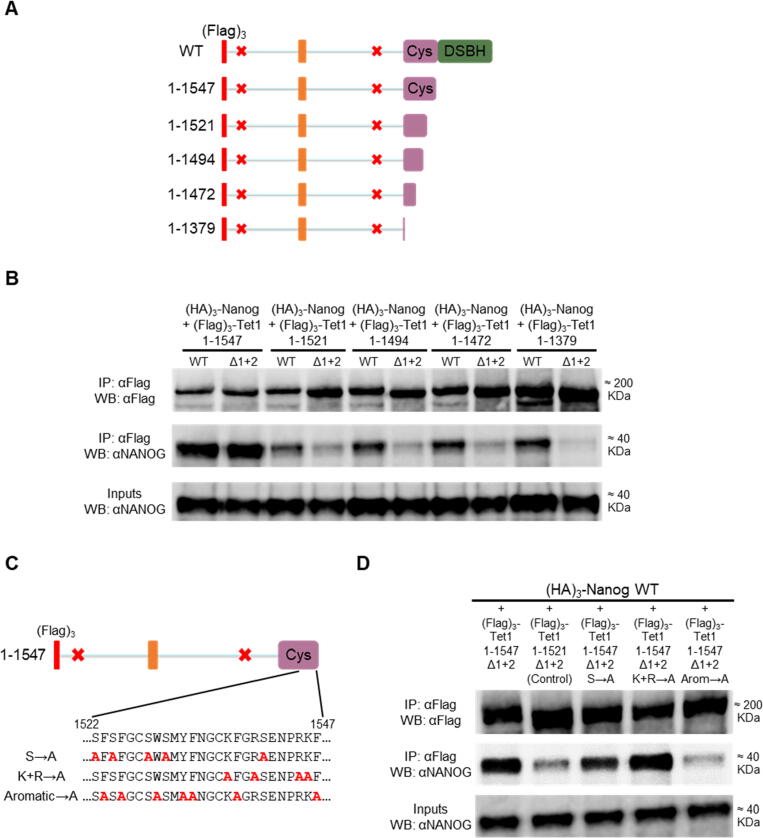
A third NANOG-interacting domain overlapping TET1 catalytic domain. (a) and (b) Co-immunoprecipitation of (Flag)_3_-TET1 truncations with (HA)_3_-NANOG in E14/T ESCs. (a) TET1 truncations were prepared in parallel in an unmutated (WT) Tet1 plasmid (not shown for simplicity) or one carrying the Δ109-120 + Δ1132-1202 (Δ1+2) mutations (red crosses). (b) Immunoblots were probed with the antibodies indicated on the left (representative images, *n* = 3). (c) and (d) Co-immunoprecipitations of (Flag)_3_-TET1 mutants with (HA)_3_-NANOG from E14/T ESCs. (c) Alanine substitution mutants between 1522 and 1547 were prepared in a plasmid expressing TET1(1–1547) with the Δ109-120 + Δ1132-1202 (Δ1+2) mutations (red crosses). (d) Immunoblots were probed with the antibodies indicated on the left (representative images, *n* = 2).

**Figure 5:**
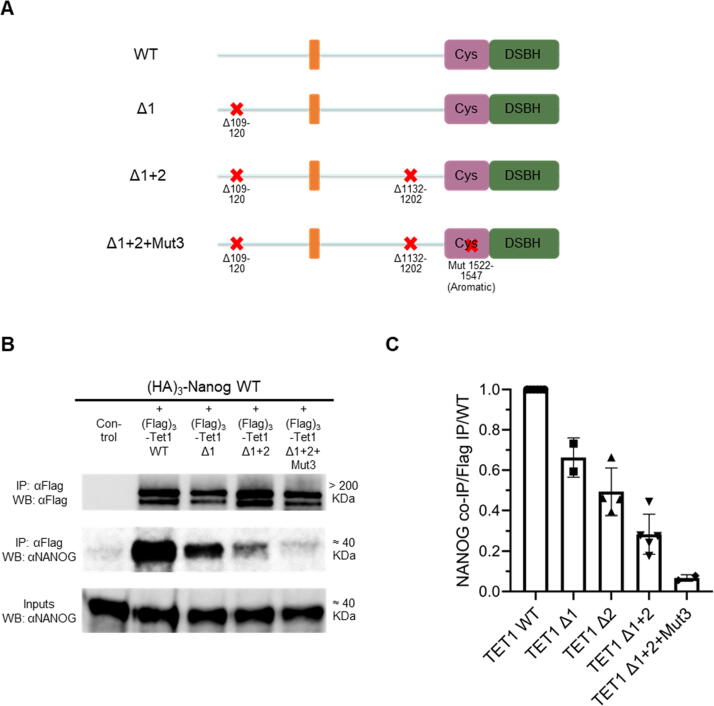
Generation of a TET1 triple-mutant unable to interact with NANOG. Co-immunoprecipitation of the indicated full-length (Flag)_3_-TET1 mutants (a) with (HA)_3_-NANOG from E14/T ESCs. (b) Immunoblots were probed with the antibodies indicated on the left (representative images, *n* = 2). (c) Co-immunoprecipitated NANOG protein was normalised to TET1 immunoprecipitation levels and expressed relative to wild-type; data points indicate independent experiments and error bars standard deviation.

The preceding results identified two independent NANOG-interacting domains within residues 109–120 (NID 1) and 1156–1169 (NID 2), respectively in the N- and C-terminal fragments. TET1 fragments containing deletions of these regions did not interact with NANOG compared to the unmutated version (Figure S4(a) and (b)). Full-length TET1 constructs with deletions in NID 1 (Δ1), NID 2 (Δ2) or both (Δ1+2) were therefore generated (Figure S4(c)). A TET1 mutant lacking a low-complexity insert (Δ1733-1901) was used as a control (Figure S4(c)), as this region has been hypothesised to function in protein–protein interactions.^37^ As expected, NANOG was co-immunoprecipitated with each of the single TET1 mutants. Surprisingly however, although the TET1 Δ1+2 double-mutant showed reduced NANOG binding compared to wild-type, binding was not completely eliminated (Figure S4(d)). This suggests that an additional NANOG-interacting domain may exist in TET1. To identify this third NID, TET1 expression plasmids were generated combining the double mutation Δ1+2 with increasing C-terminal truncations (Figure 4(a)). Plasmids with wild-type TET1 coding sequence used as controls allowed assessment of the relative importance of NIDs 1 and 2 for NANOG binding. With both wild-type and double-mutant constructs, the TET1-NANOG interaction was dramatically impaired when the TET1 C-terminus was truncated from residue 1547 to 1521 (Figure 4(b)). Smaller fragments (1–1521, 1–1494, 1–1472 and 1–1379) retained a weak residual interaction with NANOG, which was abolished in double mutants (Δ1+2). These results mapped a third NANOG-interacting domain within TET1 to residues 1522–1547 (NID 3). Most of these residues are strictly conserved in evolution as they are contained within the cysteine-rich catalytic domain (Figure S4(e)). To identify the residues within this region that bind NANOG, a TET1 construct carrying mutations in NIDs 1 and 2 (TET1 Δ1+2) was further modified by alanine substitution of serine, positively charged or aromatic residues within residues 1522–1547 (Figure 4(c)). The aromatic→A construct (F1523A, F1525A, W1529A, Y1532A, F1533A, F1538A, F1547A), but not other mutants, reduced the NANOG interaction to a similar extent as the truncated negative control (TET1 1–1521 Δ1+2) (Figure 4(d)). These data indicate that aromatic residues within TET1 1522–1547 play a critical role for interacting with NANOG in ESCs. Finally, a full-length TET1 mutant containing mutations in the three NANOG-interacting domains identified in this study (Δ109–120 + Δ1132–1202 + 1522–1547 aromatic→A) was generated and tested (Figure 5(a)). Interestingly, the sequential mutations of NIDs 1, 2 and 3 within full-length TET1 gradually decreased the interaction with NANOG, to a level comparable to the negative control (Figure 5(b) and (c)).

**Figure 6:**
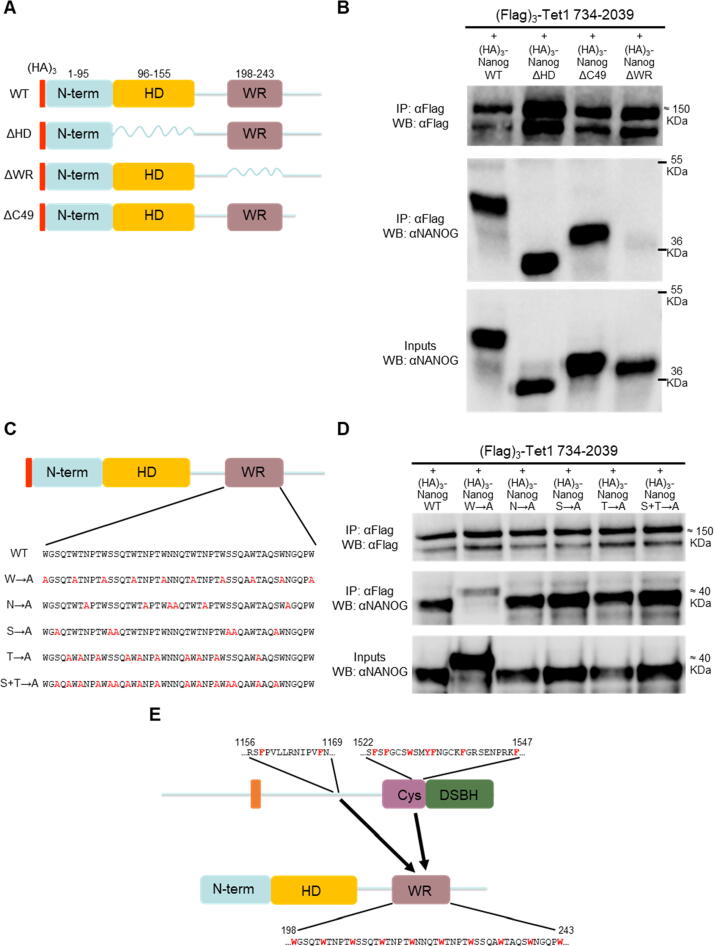
Tryptophan residues within NANOG WR interact with TET1 C-terminus. (a) and (b) Co-immunoprecipitations of (Flag)_3_-TET1 (734–2039) with the indicated (HA)_3_-NANOG deletion mutants (a) from E14/T ESCs. (b) Immunoblots were probed with the antibodies indicated on the left (representative images, *n* = 2). (c) and (d) Co-immunoprecipitation of (Flag)_3_-TET1(734–2039) with (HA)_3_-NANOG or the derivative alanine substitution mutants within the WR from E14/T ESCs. (c) Only amino acids within NANOG WR region were mutated to alanine. (d) Immunoblots were probed with the antibodies indicated on the left (representative images, *n* = 2). (e) Diagram of the interactions between the TET1 C-terminal domains (NIDs 2 and 3) and NANOG, highlighting aromatic residues critical for the interaction (red).

To identify the NANOG region(s) interacting with TET1 C-terminus, a series of (HA)_3_-tagged NANOG mutants were expressed in ESCs, together with (Flag)_3_-TET1 734–2039 (Figure 6(a)). Strikingly, only the NANOG mutant lacking the WR region (NANOG ΔWR) showed a reduced interaction with the TET1 C-terminus (Figure 6(b)). To identify residues within WR responsible for protein–protein interactions, particular amino acids (tryptophan, asparagine, serine and threonine) were substituted by alanine within the WR region of full-length NANOG (Figure 6(c)). Only the W→A mutant showed a decreased interaction with the TET1 C-terminus (Figure 6(d)), demonstrating a key role for tryptophans in the interaction of NANOG with the Tet1 C-terminus. However, full-length TET1 retained its interaction with the W→A mutant (Figure S5), confirming that other NANOG regions interact with TET1 N-terminus. Together, these experiments demonstrate that NANOG interacts with the TET1 C-terminus via aromatic residues conserved in both proteins (Figure 6(e)).

### TET1 and NANOG co-bind a subset of pluripotency enhancers associated with NANOG transcriptional target genes

**Figure 7:**
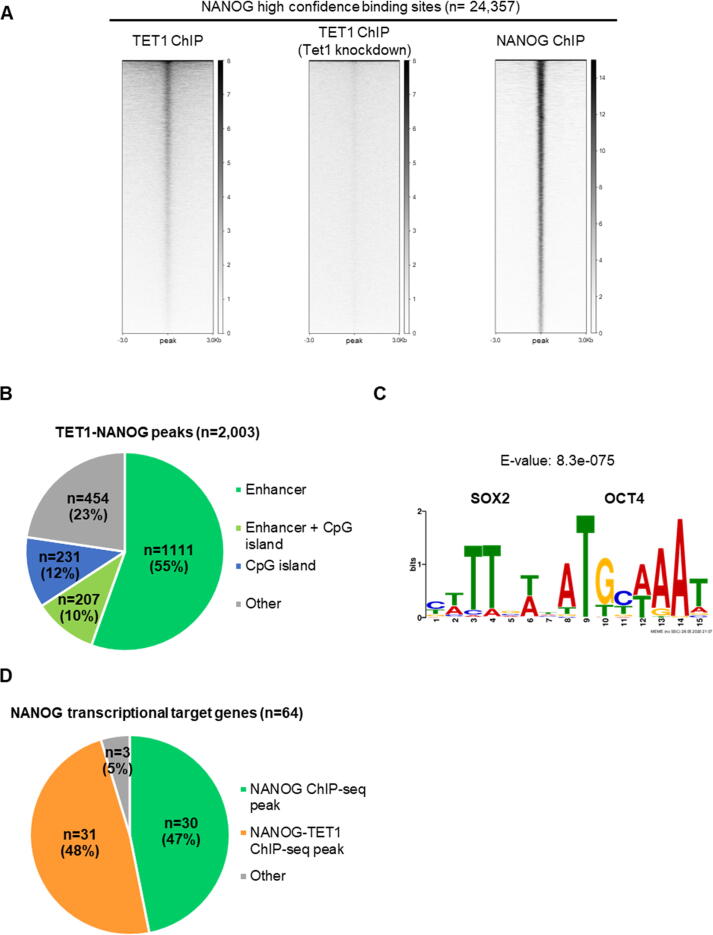
Identification of TET1-NANOG co-bound sites on chromatin in ESCs. (a) TET1 and NANOG ChIP-seq signals at NANOG “high confidence” binding sites, as defined in Figure S6(b). TET1 ChIP-seq in ESCs treated with Tet1 shRNA (knockdown) was used as a negative control. (b) Pie chart showing the portion of TET1-NANOG co-bound sites (see Figure S6(d)) overlapping with ESC enhancers and CpG islands. (c) *De novo* motif analysis performed on TET1-NANOG co-bound sites, showing the most significant binding motif and its respective E-value. (d) Pie chart showing the portion of NANOG transcriptional target genes with NANOG or TET1-NANOG co-bound sites.

Although TET1 and NANOG interact directly in ESCs, the relationship between the two proteins on chromatin remains unclear. To identify genomic sites potentially regulated by the TET1-NANOG complex, published TET1 and NANOG ChIP-seq datasets were compared. TET1 ChIP-seq peaks from two independent datasets23., 24. showed an overlap of 13,279 “high confidence” TET1 binding sites (Figure S6(a)). A similar analysis of two NANOG ChIP-seq studies38., 39. identified 24,357 “high confidence” NANOG ChIP-seq peaks (Figure S6(b)). Subsequently, TET1 and NANOG ChIP-seq signals were visualised at high confidence NANOG and TET1 binding sites, respectively. Interestingly, TET1 is enriched at the centre of a large proportion of NANOG binding sites in ESCs, and this signal is abolished upon Tet1 knockdown (Figure 7(a)). In contrast, NANOG is enriched only at a small proportion of TET1 binding sites in ESCs (Figure S6(c)). Consistent with this low level of co-enrichment, the stringent intersection of “high confidence” TET1 and NANOG ChIP-seq peaks identified only 2003 sites bound by both TET1 and NANOG (Figure S6(d) and (e)). As a first inspection, TET1-NANOG peaks were crossed with relevant genomic features, showing a large proportion of sites corresponding to ESC enhancers^40^ (65%) and a smaller proportion overlapping with CpG islands^41^ (22%) (Figure 7(b)). Remarkably, *de novo* motif analysis identified the SOX2/OCT4 composite motif at TET1-NANOG co-bound sites (Figure 7(c)). Following these observations, further analyses were performed to characterise genes associated with TET1-NANOG ChIP-seq peaks. Gene ontology analysis identified groups of genes associated with pluripotency among the top categories, such as “stem cell population maintenance”, “cellular response to leukemia inhibitory factor” and “cell fate specification” (Figure S6(f)). Importantly, TET1-NANOG ChIP-seq peaks were found within or in proximity to 48% of NANOG transcriptional target genes^42^ (Figure 7(d) and Table 1). Visual inspection of these loci showed enrichment of TET1 and NANOG ChIP-seq signals at known enhancers and putative cis-regulatory elements (Figure S6(g)). Together, these results suggest that the TET1-NANOG complex regulates a significant subset of NANOG target genes.

**Table 1:** NANOG transcriptional target genes associated with TET1-NANOG co-bound sites.

*Activated genes*	*Repressed genes*
*B4gat1*	*Ctbp2*
*Cdc42ep4*	*Dusp1*
*Cpsf4l*	*Edn2*
*En1*	*Fzd7*
*Esrrb*	*Hmces*
*Igf2bp2*	*Lefty2*
*Kit*	*Lpar6*
*Klf4*	*Nid2*
*Lmo4*	*Otx2*
*Manba*	*Raet1e*
*Mras*	*Rbm47*
*Plekhg3*	*Rbpms*
*Plpp1*	*Smagp*
*Setd1b*	*Socs3*
*Sp5*	*Tcf15*
*Tmem51*	

## Discussion

TET1^19^ and NANOG43., 44. are both expressed in the inner cell mass of the blastocyst, which is modelled *in vitro* by ESCs. TET1 and NANOG are also co-expressed in the post-implantation epiblast20., 45., 46. and in developing primordial germ cells.46., 47. Loss of either TET1 or NANOG compromises germline development.48., 49., 50., 51. Therefore, the TET1-NANOG interaction reported here may function not only at pre-implantation stages but also during later development.

Alanine substitution mutagenesis identified aromatic and hydrophobic residues that mediate the interaction between TET1 and NANOG. Tryptophan residues within the NANOG WR interact with aromatic residues in the TET1 NID 2/3, suggesting an interaction by aromatic stacking. Tryptophans within the WR are also critical for NANOG homodimerization and binding to SOX2 aromatic residues.52., 53., 30., 54. TET1 might compete with SOX2 for binding to the WR. Alternatively TET1 and other partner proteins, such as SOX2, could bind simultaneously to different residues within the WR to form larger protein complexes. The present work also demonstrates a direct, WR-independent interaction between NANOG and the TET1 N-terminus, indicative of novel protein interaction sites in NANOG.

TET1 has previously been reported to interact with the SIN3A PAH1 domain by amphipathic helix formation^35^ and with the O-linked N-acetylglucosamine transferase (OGT) via C-terminal TET1 residues.^55^ However, none of these TET1 residues overlap with the NANOG-interacting domains identified in this study. Other TET1-interacting proteins have been identified.29., 31., 32., 36., 56., 57., 58. These include thymine DNA glycosylase, which binds TET1 through at least two sites.^56^ However, apart from SIN3A and OGT, the residues mediating these interactions have not been defined. The present work demonstrates a direct physical interaction between TET1 and NANOG. Strikingly, the TET1-NANOG interaction involves multiple independent binding regions between both proteins. The three NANOG-interacting domains on TET1 (residues 109–120, 1156–1169 and 1522–1547) have not been characterised in other protein interaction studies. This adds new information about TET1 function and suggests that TET1 could also interact with other proteins through multiple binding sites. NID 1 (TET1 residues 109–120) binds NANOG in *E. coli*, indicating that the interaction is independent of post-translational modifications. However, the interaction between NID 2 (TET1 residues 1156–1169) and NANOG could not be demonstrated using a bacterial expression system. While this interaction seems to be independent of phosphorylation, other modifications like O-GlcNAcylation34., 59. might modulate this protein–protein interaction. Interestingly, NID 3 (TET1 residues 1522–1547) includes residues that interact with DNA, that lie adjacent to the TET1 catalytic domain and that are conserved in TET2 and TET3.^60^ We have recently identified two binding regions in TET2 that interact with NANOG and one of these includes residues homologous to NID 3.^21^ Importantly, this region (TET1 residues 1522–1547) also contains residues that bind methylated CpG.^60^ Therefore, binding of NANOG to NID 3 could modulate the interaction of the catalytic domain of TET1 with DNA that depends on these residues. Notably, the TET1-NANOG interaction seems to be DNA-independent since the interaction is seen in DNase-treated protein extracts, and since the interaction is unaffected by deletion of the NANOG homeodomain or the TET1 CXXC domain. It will therefore be of interest to determine whether the interaction with NANOG modulates TET1 catalytic activity.

Here, comparative analysis of TET1 and NANOG ChIP-seq datasets identified a subset of genomic loci co-bound by TET1 and NANOG in ESCs that mainly correspond to pluripotency enhancers. In contrast to most TET1 binding sites that show a broad TET1 ChIP signal, TET1 binding at TET1-NANOG co-bound sites is more narrowly focussed on NANOG peaks. Further supporting NANOG-mediated targeting to these loci, *de novo* motif analysis of these sites identified the SOX2/OCT4 motif, which characterises NANOG ChIP-seq peaks in ESCs.^61^ About half of NANOG target genes have a TET1-NANOG peak nearby, suggesting that TET1 may act cooperatively with NANOG to regulate transcription.^62^ NANOG target genes that have an associated TET1-NANOG peak include genes that are either activated or repressed by NANOG in ESCs. Potentially, TET1 could modulate transcription by demethylating enhancer DNA.18., 29., 63. Alternatively, TET1 may regulate the expression of NANOG target genes by recruiting the SIN3A co-repressor complex at these loci.23., 64., 65. However, further investigation will be required to unravel the mechanisms by which enhancers may be co-regulated by TET1 and NANOG and to distinguish action at positively and negatively regulated NANOG target genes.

## Materials and Methods

– **Molecular cloning**

Mouse TET1 open reading frame was subcloned into pPyCAG plasmids for exogenous expression of (Flag)_3_-tagged proteins under a constitutive promoter in embryonic stem cells (see Cell culture section).^43^ TET1 open reading frame was subcloned into pRSFDuet plasmids (Novagen) for exogenous expression of MBP-tagged proteins under an IPTG-inducible promoter in *E. coli* (see Preparation of protein extracts from bacterial pellets). TET1 truncations and mutants were obtained by cloning PCR products or synthetic DNA fragments (Integrated DNA Technologies, Inc.) using Gibson Assembly.^66^ For more information, please see our list of plasmid constructs, which are available upon request.

– **Cell culture**

E14/T mouse embryonic stem cells were used in this study, as they constitutively express the polyoma large T antigen and can therefore propagate pPyCAG plasmids carrying the polyoma origin of replication.^43^ ESCs were cultured in a 37 °C/7% CO_2_ incubator on gelatin-coated plates. Composition of the culture medium: Glasgow minimum essential medium (Sigma-Aldrich, cat. G5154), 10% fetal bovine serum, 1× L-glutamine (Thermo Fisher Scientific, cat. 25030024), 1× sodium pyruvate (Thermo Fisher Scientific, cat. 11360039), 1× MEM non-essential amino acids (Thermo Fisher Scientific, cat. 11140035), 0.1 mM 2-Mercaptoethanol (Thermo Fisher Scientific, cat. 31350010), 100U/ml LIF (made in-house).

To overexpress tagged proteins for co-immunoprecipitations, 3 × 10^6^ E14/T ESCs were transfected with pPyCAG plasmids of interest using Lipofectamine 3000 (Thermo Fisher Scientific, cat. L3000008). Transfections were performed in 10 cm dishes following manufacturer’s instructions. E14/T ESCs were harvested 24 h after transfection for protein extract preparation.

– **Preparation of nuclear protein extracts from embryonic stem cells**

ESCs were washed twice with PBS, trypsinised and pelleted (5 min, 400 g, 4 °C) before lysis in swelling buffer (5 mM PIPES pH8.0, 85 mM KCl) freshly supplemented with 1x protease inhibitor cocktail (Roche, cat. 04693116001) and 0.5% NP-40. After 20 min on ice with occasional shaking, nuclei were pelleted (10 min, 500 g, 4 °C) and resuspended in 1 ml of lysis buffer (20 mM HEPES pH7.6, 350 mM KCl, 0.2 mM EDTA, 1.5 mM MgCl_2_, 20% glycerol) freshly supplemented with 0.2% NP-40, 0.5 mM DTT, and 1x protease inhibitor cocktail. The material was transferred into no-stick microtubes (Alpha Laboratories, cat. LW2410AS) and supplemented with 150 U/ml of Benzonase nuclease (Millipore, cat. 71206). Samples were incubated on a rotating wheel for 30 min at 4 °C and centrifuged (16,000 g, 30 min, 4 °C) to remove any precipitate. Nuclear proteins extracts were stored at −80 °C, or used directly for immunoprecipitation or immunoblot. 30–50 µl of protein extract was used as input material and boiled in Laemmli buffer for 5 min at 95 °C.

– **Immunopurification of (Flag)_3_-tagged proteins from nuclear protein extracts**

To immunoprecipitate TET1, 5 µg of anti-Flag (Sigma-Aldrich, cat. F3165) or anti-TET1 (Millipore, cat. 09–872) antibody was added to protein extracts. For negative controls, 5 µg of normal IgG (Santa Cruz) were added to protein extracts. Samples were incubated overnight at 4 °C on a rotating wheel. 30 µl of beads coupled with ProteinA or ProteinG (GE Healthcare 4 Fast Flow Sepharose), previously blocked with 0.5 mg/ml chicken egg albumin (Sigma-Aldrich), were added to each sample, followed by a 2 h incubation at 4 °C on a rotating wheel. Beads were washed 5 times with lysis buffer (20 mM HEPES pH7.6, 350 mM KCl, 0.2 mM EDTA, 1.5 mM MgCl_2_, 20% glycerol) freshly supplemented with 0.5% NP-40 and 0.5 mM DTT. Between each wash, samples were centrifuged at 4 °C for 1 min at 2,000 rpm. After the final wash, beads were resuspended in Laemmli buffer and boiled for 5 min at 95 °C

As an alternative strategy to immunoprecipitate (Flag)_3_-tagged proteins, 30 µl of anti-Flag magnetic beads (Sigma-Aldrich, cat. M8823) was added to each protein extract. To immunoprecipitate endogenous TET1 from *Tet1-(Flag)_3_* ESCs, 150 µl of anti-Flag magnetic beads were added to nuclear protein extracts obtained from ≈200 million cells, as described above. Samples were incubated on a rotating wheel for 2 h at room temperature. Following three washes with PBS using a magnet (Thermo Fisher Scientific, cat. 12321D), magnetic beads were resuspended in Laemmli buffer and boiled for 5 min at 95 °C. Samples were stored at −20 °C or analysed directly by immunoblot.

– **Preparation of protein extracts from bacterial pellets**

Chemically competent BL21(DE3) *E. coli* (NEB, cat. C2527I) were transformed with pRSF bacterial expression plasmids of interest. A single colony was inoculated in LB medium supplemented with appropriate antibiotics and incubated overnight in a 37 °C shaker (225 rpm). The overnight culture was diluted (1/50) in a 50 ml flask containing 50 ml of LB medium supplemented with appropriate antibiotics and incubated in a 37 °C shaker (225 rpm) until the culture reached the exponential phase (≈3 h, *A*_600_: 0.5–0.7). 1 mM IPTG was added to the culture to initiate protein expression, and cells were transferred in an 18 °C shaker (225 rpm) for 6 h. Bacterial pellets were collected by centrifugation (5000 g, 10 min) and stored at −20 °C until protein extraction.

To prepare protein extracts, bacterial pellets were resuspended in 5 ml of cold protein extraction buffer (25 mM Tris–HCl pH 8.0, 200 mM NaCl), and sonicated 3x1 min on ice. Samples were centrifuged (16,000 g, 30 min, 4 °C) to remove insoluble material. Bacterial protein extracts were stored at 4 °C or used directly for protein purification. 30–50 µl of protein extract was used as input material and boiled in Laemmli buffer for 5 min at 95 °C.

– **Purification of MBP-tagged proteins from bacterial extracts**

To purify MBP-tagged proteins, each bacterial protein extract was loaded into a gravity flow column containing 600 µl of amylose resin. The resin was washed once with cold protein extraction buffer (25 mM Tris–HCl pH 8.0, 200 mM NaCl) and MBP-tagged proteins were eluted in 500 µl of cold protein extraction buffer (25 mM Tris–HCl pH 8.0, 200 mM NaCl) supplemented with 10 mM Maltose. 50 µl of eluate was boiled in Laemmli buffer for 5 min at 95 °C.

– **Immunoblot**

Protein samples were loaded into Bolt 10% Bis–Tris Plus Gels (Thermo Fisher Scientific, cat. NW00102BOX) with 1× Bolt MOPS SDS running buffer (Thermo Fisher Scientific, cat. B0001). 10 µl of SeeBlue Plus2 pre-stained protein standard (Thermo Fisher Scientific, cat. LC5925) was used to visualize protein molecular weight. The electrophoresis was performed at 160 V for 1 h. Proteins were transferred overnight at 4 °C onto a nitrocellulose membrane (150 mA constant current) with transfer buffer (25 mM Tris, 0.21 M glycine, 10% methanol). The membrane was blocked for 1 h at room temperature with 10% (w/v) non-fat skimmed milk dissolved in PBS supplemented with 0.1% Tween. Then, the membrane was incubated for 1 h at room temperature with primary antibodies diluted to the working concentration in 5% (w/v) non-fat skimmed milk dissolved in PBS supplemented with 0.1% Tween. The membrane was washed 3 times with PBS supplemented with 0.1% Tween, and incubated for 2 h at room temperature with LI-COR IRDye conjugated secondary antibodies diluted 1:5,000 in 5% non-fat skimmed milk dissolved in PBS supplemented with 0.1% Tween. The membrane was finally washed 3 times with PBS supplemented with 0.1% Tween and analysed using the auto-scan function of the LI-COR Odyssey FC imaging system. Molecular weights of protein bands were evaluated by visual comparison with fluorescent protein standards (SeeBlue Plus2 Pre-stained ladder, Thermo Fisher Scientific, cat. LC5925; or Chameleon Duo Pre-stained ladder LI-COR, cat. 928–60000). For protein quantification, the relevant bands were quantified using the LI-COR Image Studio Software.

– **Antibodies**

**Table:** 

**Antibody**	**Reference**	**Working dilution (immunoblot)**
Flag	Sigma-Aldrich, cat. F3165	1:5,000
HA	Covance, cat. MMS-101P	1:5,000
Nanog	Bethyl, cat. A300-397A	1:2,000
GST	Abcam, cat. ab92	1:2,000
His tag	Abcam, cat. ab18184	1:2,000
MBP	NEB, cat. E8032S	1:10,000
Anti-mouse (secondary)	LI-COR, cat. 926-68072	1:5,000
Anti-rabbit (secondary)	LI-COR, cat. 926-32213	1:5,000

– **Protein alignments**

To identify evolutionary conserved residues, TET1 protein sequences from various mammalian and non-mammalian species were downloaded from UNIPROT (https://www.uniprot.org/) and aligned using ESPript (http://espript.ibcp.fr).^67^

– **ChIP-seq analysis**

ChIP-seq datasets were analysed using the Galaxy platform (https://usegalaxy.org).^68^ Details concerning the bioinformatic workflow are available at the following address: https://usegalaxy.org/u/raf4579/w/workflow-chip-seq-1. Raw sequencing data (FASTQ files) was downloaded from publicly available databases NCBI's Gene Expression Omnibus or ArrayExpress. Quality control was performed using the software “FastQC” (Babraham Bioinformatics). Samples were filtered to remove contaminating adapter sequences and low-quality reads (cut-off quality score >20.0). Reads were mapped to the mouse mm9 reference genome using “Bowtie2” (BAM file output).^69^ Reads were mapped only to a unique genomic location (*k* = 1). ChIP-seq peaks were called using the software “MACS2” (BED file output).^70^ The immunoprecipitated sample was compared to the genomic input for identifying statistically significant binding sites (qvalue 0.05). For the analysis of NANOG ChIP-seq datasets, the algorithm optimised for “narrow peaks” was used. For the analysis of TET1 ChIP-seq datasets, the algorithm optimised for “broad peaks” was used. If replicates were available, only ChIP-seq peaks shared between replicates were considered for further analyses. Peaks were considered as shared between datasets when presenting an overlap of at least 1 bp. To visualise ChIP-seq datasets on a genome browser, mapped reads (BAM files) from TET1 ChIP-seq with (GSM611195) or without (GSM611194) Tet1 knockdown, as well as NANOG ChIP-seq (GSM1082342) were converted into bigWig files using “Deeptools”.^71^ Data was normalised in “Reads Per Kilobase Million” (RPKM) to allow the comparison between ChIP-seq datasets. Genomic snapshots were taken using the genome viewer “IGV”.^72^ To visualise ChIP-seq datasets as heatmaps, the software “Deeptools” was used.^71^ To perform *de novo* motif analysis on ChIP-seq datasets, the DNA sequences corresponding to each ChIP-seq peak were extracted (FASTA file output) and analysed using the “MEME” software.^73^ Motifs between 5 and 25 bp, enriched with a *E* value <0.05, were identified. These results were compared to known protein motifs in the JASPAR database.^74^ ChIP-seq peaks were assigned putative target genes using the “Genomic Regions Enrichment of Annotations Tool” (GREAT v4.04: http://bejerano.stanford.edu/great/public/html/index.php) with default parameters (basal regulatory domain extending 5 kb upstream and 1 kb downstream from transcription start site, and an extension up to the basal regulatory domain of the nearest upstream and down-stream genes within 1 Mb). Gene ontology analysis from ChIP-seq peaks was performed using the “Genomic Regions Enrichment of Annotations Tool” (GREAT v4.04: http://bejerano.stanford.edu/great/public/html/index.php).

## Accession numbers

UniProt Knowledgebase (UniProtKB) accession numbers: **E9Q9Y4** (Mouse TET1 protein sequence), **Q80Z64** (Mouse NANOG protein sequence).

NCBI Gene Expression Omnibus accession numbers: **GSE24841** (TET1 ChIP-seq), **GSE26832** (TET1 ChIP-seq), **GSE44286** (NANOG ChIP-seq).

ArrayExpress accession numbers: **E-MTAB-1617** (NANOG ChIP-seq).

## CRediT authorship contribution statement

**Raphaël Pantier:** Conceptualization, Methodology, Investigation, Writing - original draft, Writing - review & editing, Visualization. **Nicholas Mullin:** Conceptualization, Methodology, Supervision, Writing - review & editing. **Elisa Hall-Ponsele:** Investigation. **Ian Chambers:** Conceptualization, Writing - original draft, Writing - review & editing, Supervision, Project administration, Funding acquisition.
