# Supplementary material for: TET1 Interacts Directly with NANOG via Independent Domains Containing Hydrophobic and Aromatic Residues
Source: J Mol Biol. 2020 Nov 20;432(23):6075–91. doi: 10.1016/j.jmb.2020.10.008 (PMC7763487; doi:10.1016/j.jmb.2020.10.008)
Supplement: Supplementary data 1 [file mmc1.docx]

**Supplementary information**

**
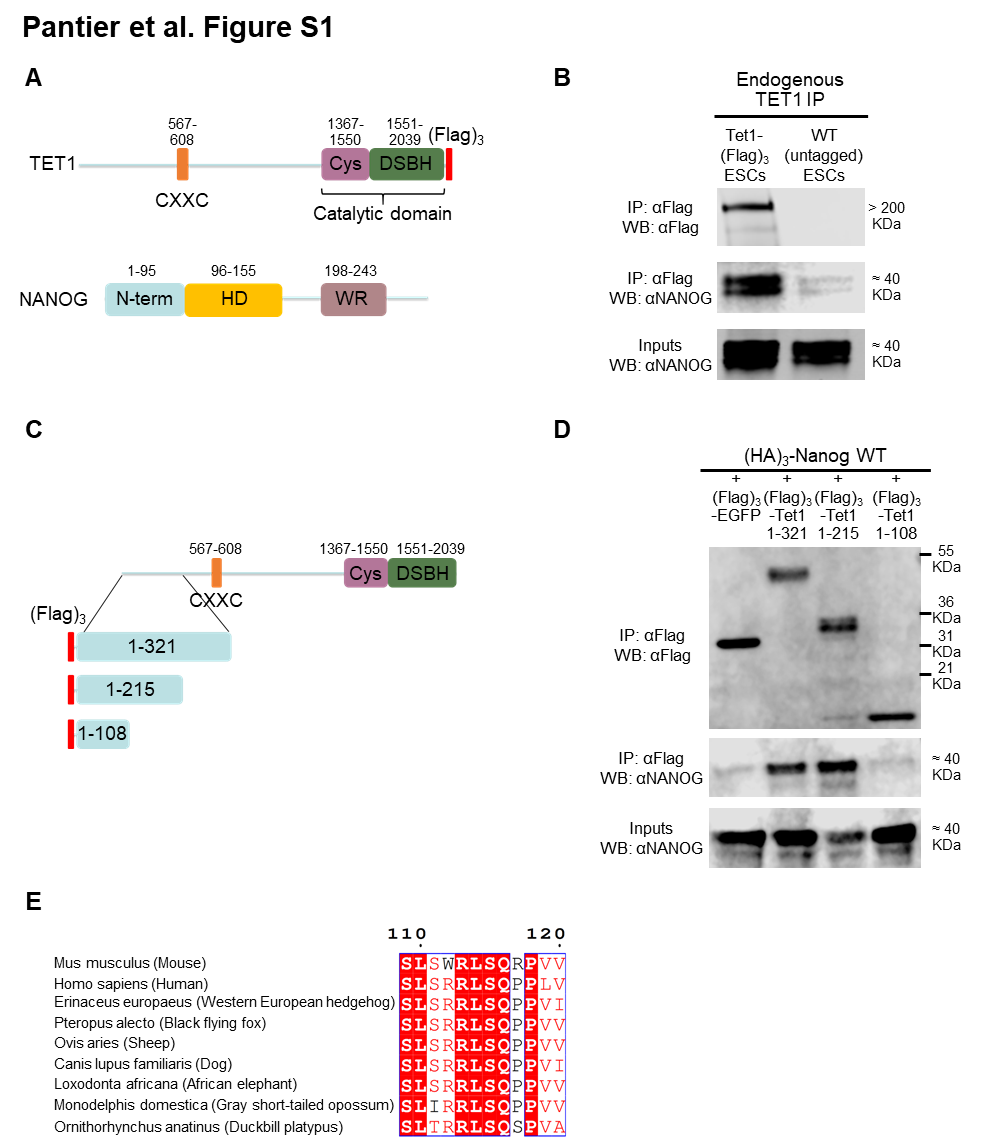
**

**Supplementary Figure 1 (related to Figure 1)**

**A.** Diagrammatic representation of TET1 and NANOG primary structures; numbers indicate amino acid residues. **B.** Co-immunoprecipitation of endogenously expressed TET1-(Flag)_3_ and NANOG from *Tet1-(Flag)_3_* homozygous ESCs [21]. Immunoblots were probed with the antibodies indicated on the left (representative images, n=3). **C, D.** Co-immunoprecipitations of (Flag)_3_-TET1 N-terminal constructs with (HA)_3_-NANOG from E14/T ESCs. **C**, Fragments of the TET1 N-terminus are shown in the context of full length TET1. **D**, Immunoblots were probed with the antibodies indicated on the left; (Flag)_3_-EGFP was used as a negative control (representative images, n=2). **E.** Alignment of the NANOG-interacting domain 1 (NID 1) in mammalian TET1 proteins, centred on mouse TET1 residues (109-120). Identical residues are white on a red background; conservative substitutions found in several mammalian species are in red.


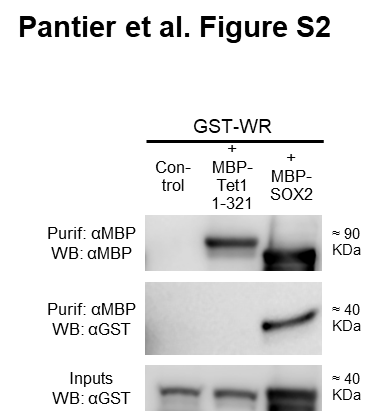


**Supplementary Figure 2 (related to Figure 2)**

Analysis of MBP complexes of MBP-TET1 (1-321) or MBP-SOX2 (positive control, [30]) for the presence of GST-WR. Immunoblots were probed with the antibodies indicated on the left (representative images, n=2).


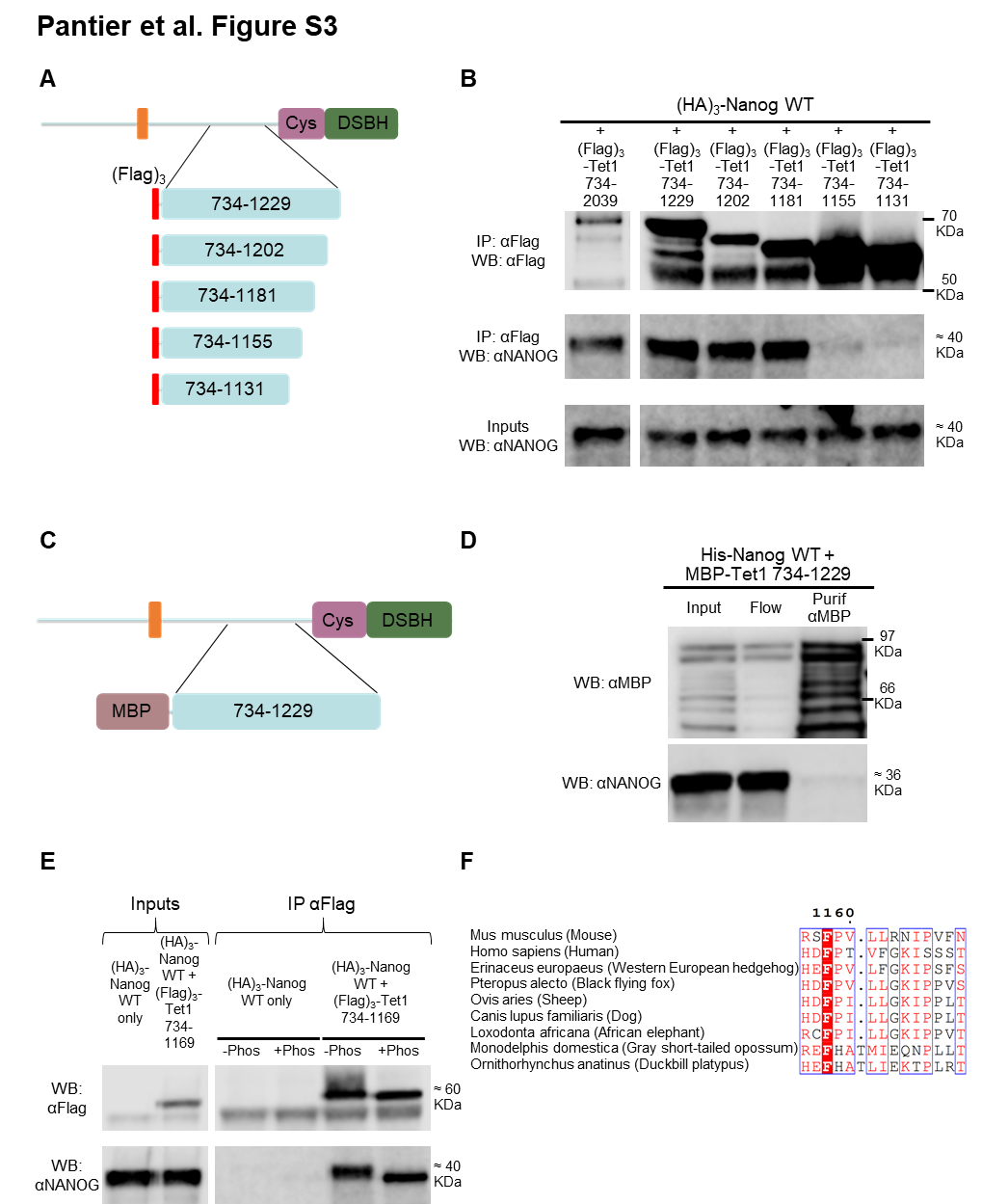


**Supplementary Figure 3 (related to Figure 3)**

**A, B.** Co-immunoprecipitation of C-terminal truncations of (Flag)_3_-TET1(734-1229) (**A**) with (HA)_3_-NANOG from E14/T ESCs. **B**, Immunoblots were probed with the antibodies indicated on the left (representative images, n=3). **C, D.** Co-purification of MBP-TET1(734-1229) (**C**) with His-NANOG from *E.coli*. **D**, Immunoblots were probed with the antibodies indicated on the left (representative images, n=2). **E.** Co-immunoprecipitations of (Flag)_3_-TET1(734-1169) with (HA)_3_-NANOG from E14/T ESCs with (+Phos) or without (-Phos) phosphatase treatment. Immunoblots were probed with the antibodies indicated on the left. **F.** Alignment of the NANOG-interacting domain 2 (NID 2) in mammalian TET1 proteins, centred on mouse TET1 residues (1156-1169). Identical residues are white on a red background; conservative substitutions found in several mammalian species are in red.


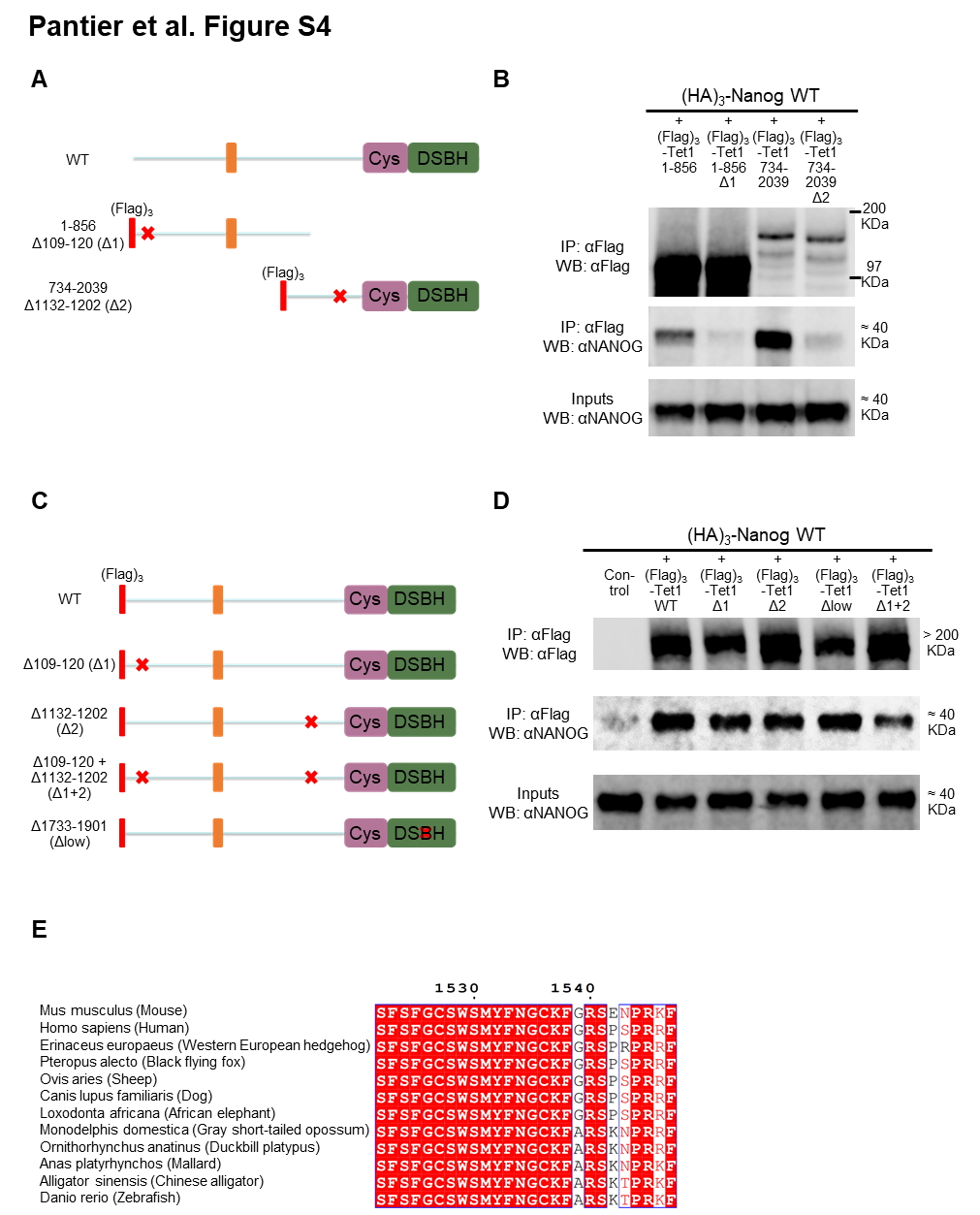


**Supplementary Figure 4 (related to Figure 4)**

**A, B.** Co-immunoprecipitation of (Flag)_3_-TET1 truncations (**A**) with (HA)_3_-NANOG from E14/T ESCs. TET1 truncations were prepared in plasmids carrying the Δ109-120 (Δ1) or Δ1132-1202 (Δ2) mutations indicated (red crosses). **B**, Immunoblots were probed with the antibodies indicated on the left (representative images, n=3). **C, D.** Co-immunoprecipitation of full-length (Flag)_3_-TET1 mutants (**C**) with (HA)_3_-NANOG in E14/T ESCs. TET1 constructs carried the Δ109-120 (Δ1), Δ1132-1202 (Δ2) or Δ1733-1901 (Δlow) mutations indicated (red crosses). **D**, Immunoblots were probed with the antibodies indicated on the left (representative images, n=3). **E.** Alignment of the NANOG-interacting domain 3 (NID 3) of TET1 from the indicated species, centred on mouse TET1 residues (1522-1547). Identical residues are white on a red background; conservative substitutions found in several mammalian species are in red.


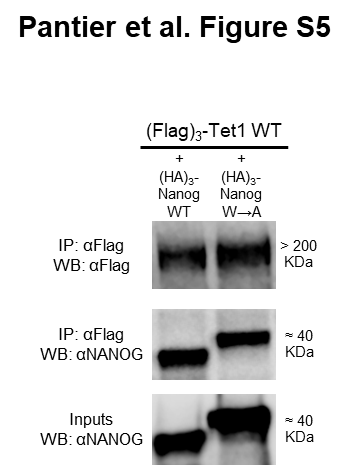


**Supplementary Figure 5 (related to Figure 6)**

Co-immunoprecipitation of full-length (Flag)_3_-TET1 WT with (HA)_3_-NANOG W→A (in which all 10 tryptophan residues within the WR were substituted by alanine; see Figure 6C) in E14/T ESCs. Immunoblots were probed with the antibodies indicated on the left (representative images, n=3).


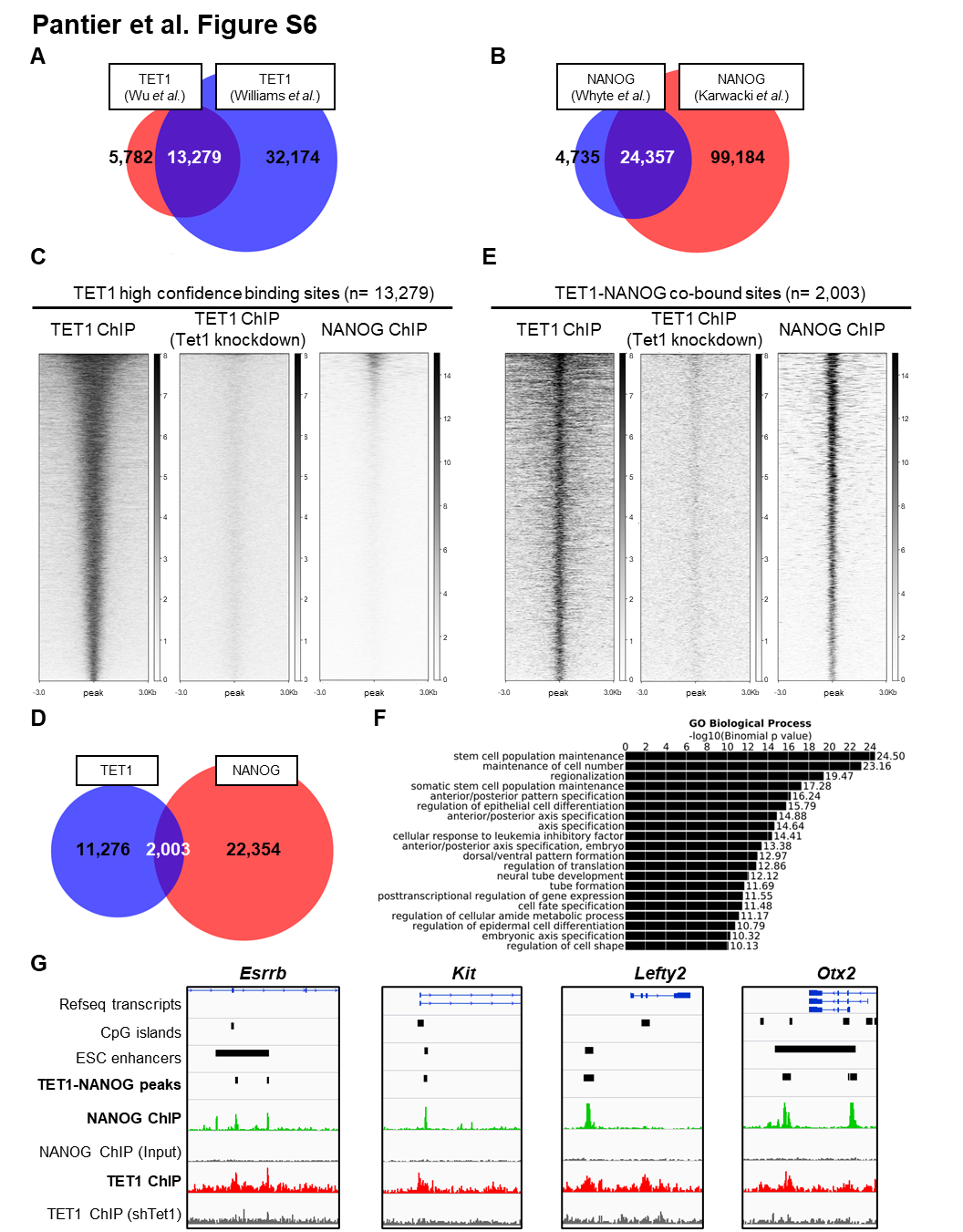


**Supplementary Figure 6 (related to Figure 7)**

**A.** Venn diagram showing the overlap of TET1 ChIP-seq peaks between two published datasets in mouse ESCs [23,24]. High confidence TET1 binding sites are shared between datasets (n= 13,279). **B.** Venn diagram showing the overlap of NANOG ChIP-seq peaks between two published datasets in mouse ESCs [38,39]. High confidence NANOG binding sites are shared between datasets (n= 24,357). **C.** TET1 and NANOG ChIP-seq signal at TET1 “high confidence” binding sites, as defined in Figure S6A. TET1 ChIP-seq in ESCs treated with Tet1 shRNA (knockdown) was used as a negative control. **D.** Venn diagram showing the overlap of TET1 (blue) and NANOG (red) high confidence ChIP seq peaks in mouse ESCs. **E.** TET1 and NANOG ChIP-seq signal at TET1-NANOG co-bound sites, as defined in Figure S6D. TET1 ChIP-seq in ESCs treated with Tet1 shRNA (knockdown) was used as a negative control. **F.** Gene ontology analysis performed on genes associated with TET1-NANOG co-bound sites. **G.** Genomic snapshots showing NANOG (green) and TET1 (red) ChIP-seq signals in the vicinity of NANOG transcriptional target genes.
